# Supplementary material for: Taxonomic revision of the tarantula genus Aphonopelma Pocock, 1901 (Araneae, Mygalomorphae, Theraphosidae) within the United States
Source: Zookeys. 2016 Feb 4;(560):1–340. doi: 10.3897/zookeys.560.6264 (PMC4768370; doi:10.3897/zookeys.560.6264)
Supplement: Supplementary material 2 — Compressed files (.zip) containing Aphonopelma boxplot and PCA morphospace data. [file zookeys-560-001-s002.zip › Supp2a.zip!/Appendix 2A/PCA_morphospace/Aphonopelma_PCA_females/ALL/females_all_PC2~PC3.pdf]

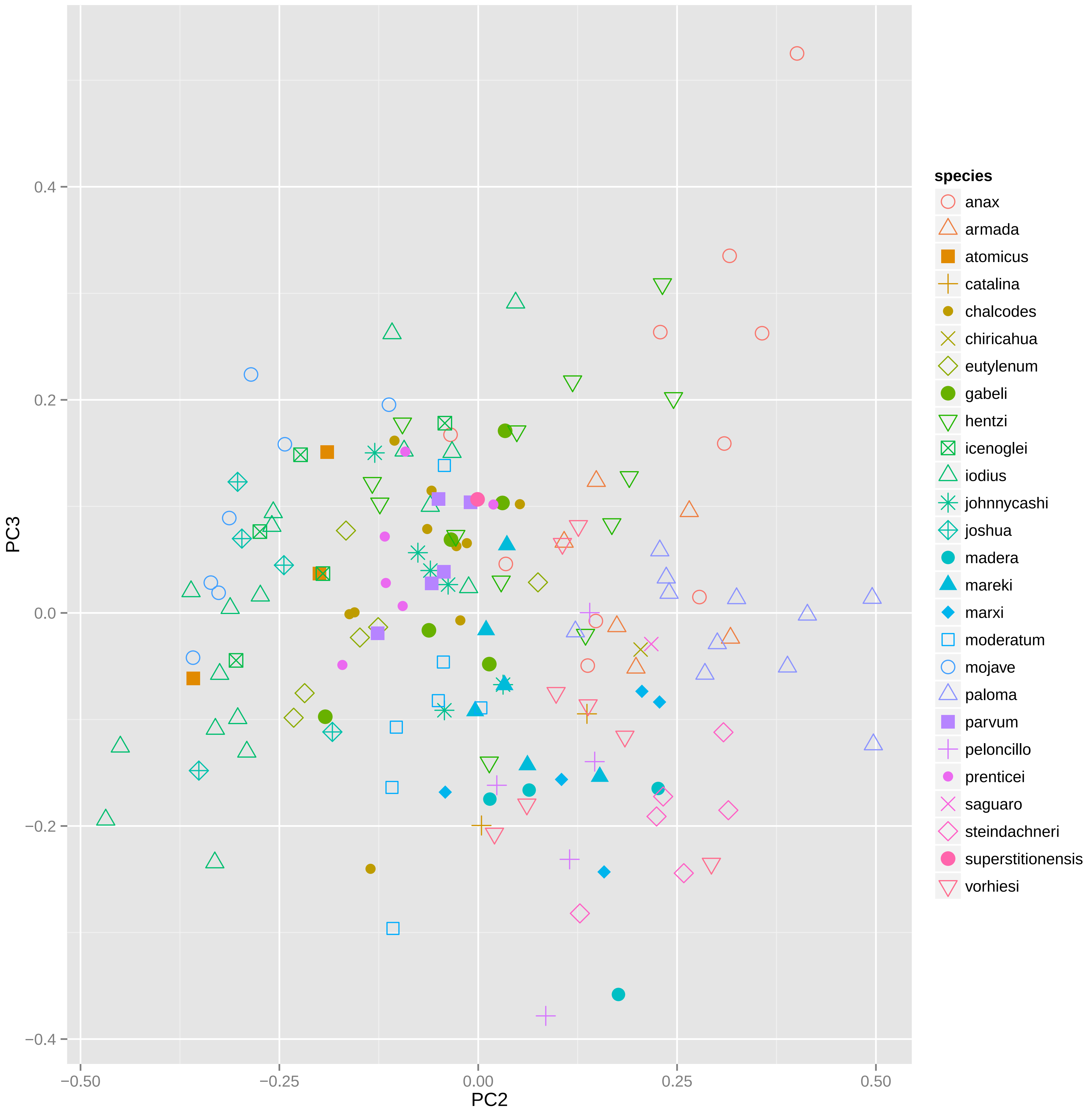

PC3
PC2
0.4
0.2
0.0
−0.2
−0.4
−0.50
−0.25
0.00
0.25
0.50
species
anax
armada
atomicus
catalina
chalcodes
chiricahua
eutylenum
gabeli
hentzi
icenoglei
iodius
johnnycashi
joshua
madera
mareki
marxi
moderatum
mojave
paloma
parvum
peloncillo
prenticei
saguaro
steindachneri
superstitionensis
vorhiesi
